# Inheritance and Bulked Segregant Analysis of Leaf Rust and Stem Rust Resistance in Durum Wheat Genotypes

**DOI:** 10.1094/PHYTO-12-16-0444-R

**Published:** 2017-09-29

**Authors:** Meriem Aoun, James A. Kolmer, Matthew N. Rouse, Shiaoman Chao, Worku Denbel Bulbula, Elias M. Elias, Maricelis Acevedo

**Affiliations:** Department of Plant Pathology, North Dakota State University, Fargo 58108; United States Department of Agriculture–Agricultural Research Service (USDA-ARS), Cereal Disease Laboratory, and Department of Plant Pathology, University of Minnesota, St. Paul 55108; United States Department of Agriculture–Agricultural Research Service (USDA-ARS), Cereal Disease Laboratory, and Department of Plant Pathology, University of Minnesota, St. Paul 55108; USDA-ARS Genotyping Laboratory, Biosciences Research Laboratory, Fargo, ND, 58102; Ethiopian Institute of Agricultural Research, P.O. Box 32, Debre Zeit, Ethiopia; Department of Plant Sciences, North Dakota State University; International Programs, College of Agriculture and Life Sciences, Cornell University, Ithaca, NY

## Abstract

Leaf rust, caused by *Puccinia triticina*, and stem rust, caused by *P. graminis* f. sp. *tritici*, are important diseases of durum wheat. This study determined the inheritance and genomic locations of leaf rust resistance (*Lr*) genes to *P. triticina* race BBBQJ and stem rust resistance (*Sr*) genes to *P. graminis* f. sp. *tritici* race TTKSK in durum accessions. Eight leaf-rust-resistant genotypes were used to develop biparental populations. Accessions PI 192051 and PI 534304 were also resistant to *P. graminis* f. sp. *tritici* race TTKSK. The resulting progenies were phenotyped for leaf rust and stem rust response at seedling stage. The *Lr* and *Sr* genes were mapped in five populations using single-nucleotide polymorphisms and bulked segregant analysis. Five leaf-rust-resistant genotypes carried single domi-nant *Lr* genes whereas, in the remaining accessions, there was deviation from the expected segregation ratio of a single dominant *Lr* gene. Seven genotypes carried *Lr* genes different from those previously characterized in durum. The single dominant *Lr* genes in PI 209274, PI 244061, PI387263, and PI 313096 were mapped to chromosome arms 6BS, 2BS, 6BL, and 6BS, respectively. The *Sr* gene in PI 534304 mapped to 6AL and is most likely *Sr13*, while the *Sr* gene in PI 192051 could be uncharacterized in durum.

Durum wheat (*Triticum turgidum* L. var. *durum* (Desf.)), an allo-tetraploid (2n = 4x = 28), is economically an important cereal crop used primarily for pasta production. Durum wheat is grown mainly in the Mediterranean countries, Canada, Mexico, the United States, and Ethiopia (Goyeau et al. 2012; Habash et al. 2009; Ordon˜ez and Kolmer 2007b; Vavilov 1951). North Dakota is the largest durum-producing state in the United States, accounting for more than 50% of the total U.S. production, which is worth more than $300 million per year (NASS 2016).

Wheat rust diseases have historically been a major constraint for wheat production, severely reducing yield and kernel quality. Durum wheat has been traditionally considered more resistant to leaf rust (caused by *Puccinia triticina* Erikss.) than common wheat (*T. aestivum* L.; 2n = 6x = 42). However, in recent years, *P. triticina* races highly virulent on resistant durum wheat cultivars are increasingly affecting durum production worldwide (Goyeau et al. 2006; Huerta-Espino et al. 2009; Singh et al. 2004). For instance, *P. triticina* race BBG/BN and its variants, with virulence to leaf rust resistance (*Lr*) gene *Lr72,* overcame the resistance of the adapted CIMMYT durum wheat cultivars in northwestern Mexico, which resulted in severe yield losses (Huerta-Espino et al. 2011; Singh et al. 2004). Similarly, increased susceptibility of durum wheat cul-tivars to leaf rust occurred in other durum-producing areas, including the Mediterranean basin, the Middle East, and Chile (Goyeau et al. 2012; Martinez et al. 2005; Ordon˜ez and Kolmer 2007a; Singh et al. 2004). In the United States, a race with a virulence phenotype and simple sequence repeat (SSR) genotype similar to the previ-ously identified BBG/BN Mexican race was collected on durum in California in 2009 (Kolmer 2013). This race was designated as BBBQJ following the *P. triticina* nomenclature system of Long and Kolmer (1989). The same race was later collected in 2013 on ‘Overley’ hard red winter wheat in Kansas (Kolmer 2015). This race is also virulent to *Lr39/41* that is present in many hard red winter wheat cultivars grown in the Southern Great Plains. This race could become established in the winter wheat crop and then migrate northward to the durum producing region of North Dakota (Kolmer 2015).

Typically, the *P. triticina* isolates virulent on durum wheat cul-tivars are different in their virulence phenotypes from the common wheat-type isolates because these are avirulent to many of the *Lr* genes present in common wheat (Goyeau et al. 2006; Ordon˜ez and Kolmer 2007a). The *P. triticina* isolates collected from common wheat are generally avirulent on durum wheat (Huerta-Espino and Roelfs 1992; Ordon˜ez and Kolmer 2007a; Singh 1991). Currently, few *Lr* genes have been mapped in durum wheat. Characterized *Lr* genes in durum and other tetraploid wheat subspecies include *Lr3a* (Herrera-Foessel et al. 2005), *Lr10* (Aguilar-Rincon et al. 2001), *Lr14a* (Herrera-Foessel et al. 2008b), *Lr23* (McIntosh and Dyck 1975; Nelson et al. 1997), the complementary gene pair *Lr27*+*31* (Singh and McIntosh 1984; Singh et al. 1993), *Lr33* (Dyck 1994; Dyck et al. 1987), *Lr46* (Herrera-Foessel et al. 2011), *Lr47* (Dubcovsky et al. 1998), *Lr52* (Singh et al. 2010), *Lr61* (Herrera-Foessel et al. 2008a), *Lr64* (Dyck 1994; McIntosh et al. 2009), *Lr72* (Herrera-Foessel et al. 2014a), and *LrCamayo* (Herrera-Foessel et al. 2007). However, races with virulence to most of these *Lr* genes are currently present. For instance, virulence to *Lr10*, *Lr23*, and *Lr33* is common in durum-type *P. triticina* races (Huerta-Espino and Roelfs 1992; Ordon˜ez and Kolmer 2007a; Singh et al. 2005). In addition, *P. triticina* race BBG/ BN and its variants are virulent to *Lr72* (Huerta-Espino et al. 2011; Singh et al. 2004). A *P. triticina* race virulent to *Lr27*+*Lr31* and *Lr3a* was detected in Mexico in 2008 (Huerta-Espino et al. 2009). Similarly, a race of *P. triticina* that was collected in Mexico in 2010 is virulent to *Lr61* (Herrera-Foessel et al. 2014b). The gene *Lr14a* is not effective against the common races currently present in France, Spain, Chile, Argentina, Morocco, and Tunisia (Gharbi et al. 2013; Goyeau et al. 2012; Ordon˜ez and Kolmer 2007a; Soleiman et al. 2016) (J. A. Kolmer and M. Acevedo, unpublished). Therefore, the identification of new *Lr* genes is crucial to mitigate durum wheat yield loss caused by leaf rust.

Stem rust, caused by *P. graminis* f. sp. *tritici* Erikss. & Henning, is one of the most destructive diseases of common wheat and durum wheat that can result in a complete loss of the crop under high disease severity (McIntosh and Brown 1997; Singh et al. 2011). The race TTKSK (Ug99) was first detected in Uganda in 1998 (Pretorius et al. 2000). This race spread to Kenya in 2001 and to Ethiopia by 2003. It was later detected in Sudan, Yemen, Iran, South Africa, and Egypt (Jin et al. 2008; Nazari et al. 2009; Pretorius et al. 2010; RustTracker. org 2016). Currently, more than 60 stem rust resistance (*Sr*) genes have been identified in wheat (McIntosh et al. 2013, 2014; Rahmatov et al. 2016) and approximately 29 are effective to races of the Ug99 race group (Niu et al. 2014; Yu et al. 2014; Yu et al. 2015). However, the resistance levels conferred by these *Sr* genes differ. For instance, only a few of these Ug99-effective *Sr* genes are effective to a broad spectrum of other *P. graminis* f. sp. *tritici* races (Singh et al. 2015; Yu et al. 2014). In addition, many of these *Sr* genes were transferred to wheat from wild relatives, thus reducing the linkage drag associated with the alien translocations carrying the genes is required before using these resistance sources in breeding lines (Singh et al. 2011, 2015).

In durum wheat, the mapped *Sr* genes and quantitative trait loci associated with stem rust resistance are limited compared with those mapped in common wheat. The resistance to race TTKSK in durum wheat, particularly in the North American cultivars, is mainly due to the presence of *Sr13* originating from the emmer wheat (*T. turgidum* L. subsp. *dicoccum*) ‘Khapli’ (Jin et al. 2007; Klindworth et al. 2007). However, in recent years, *P. graminis* f. sp. *tritici* races different from the Ug99 lineage group (TRTTF and JRCQC) have been identified in Ethiopia with combined virulence on *Sr13* and *Sr9e* (Olivera et al. 2012, 2015). Therefore, widening the global genetic diversity of stem rust resistance in durum wheat germplasm is urgently required for more durable resistance.

Whereas quantitative adult plant resistance, often based on several minor alleles or genes (Gustafson and Shaner 1982), is a very im-portant objective in breeding programs, pyramiding several qualitative resistance genes that can be identified at the seedling stage is another approach to achieve durable resistance. Seedling tests allow for screening many lines in a short period of time and small space com-pared with adult-plant tests in field trials (Letta et al. 2014).

The use of biparental mapping populations has been the standard approach to identify the chromosomal locations of plant disease resistance loci. Bulked segregant analysis (BSA) is a quick and relatively inexpensive method to efficiently identify molecular markers associated with a trait response. The procedure consists of comparing two pooled DNA samples of individuals from a segregating population originating froma single cross.Within each bulk, the individuals are identical for the trait or gene of interest but are segregating randomly for all other genes. The two bulks that are contrasting for a trait such as response to a disease are analyzed to find molecular markers that differentiate them. Therefore, the markers that are polymorphic between the pools will be linked genetically to the locus that is associated with the trait used to make the bulk (Michelmore et al. 1991).

The objective of the current studywas to determine the inheritance of leaf rust (P. triticina race BBBQJ) and stemrust (P. graminis f. sp. tritici race TTKSK) resistance at the seedling stage in eight durum wheat genotypes selected from the United States Department of Agriculture (USDA) National Small Grains Collection (NSGC), Aberdeen, ID. Genomic regions of the Lr and Sr genes weremapped in five biparental populations using high-density single-nucleotide polymorphism (SNP) markers and the BSA approach.

## Materials And Methods

**Biparental crosses and characterization of leaf rust resistance inheritance**. Eight resistant genotypes were selected from the USDA-NSGC for their low infection types to *P. triticina* race BBBQJ to develop biparental populations (Table 1). These ge-notypes were plant introduction (PI) 534304, PI 313096, PI 387263, PI 209274, PI 278379, PI 244061, PI 192051, and PI 195693. These genotypes were previously reported to carry resistance to several *P. triticina* races at the seedling stage in the greenhouse and at the adult-plant stage in the field in several locations worldwide (Aoun et al. 2016). These resistant parental lines were originally collected from Ethiopia, Portugal, Cyprus, Australia, Malta, and Yemen. All of these genotypes are landraces, except for PI 209274, which is a breeding line. The susceptible parents of the crosses were ‘Rusty’ or ‘Divide’. Divide was released in 2005 by North Dakota State Uni-versity (NDSU) and currently occupies approximately 30% of the total durum wheat acreage in North Dakota (NASS 2015). The rust-susceptible line Rusty (registration number GS-155, PI 639869) was released in 2004 by the USDA Agricultural Research Service (ARS) Northern Crops Science Laboratory, Fargo, ND and NDSU (Klindworth et al. 2006).

Crosses between resistant and susceptible parents were made at the North Dakota Agricultural Experiment Station Greenhouse Complex, Fargo, during summer 2013. In all of the biparental pop-ulations, Rusty and Divide were the female parents of the crosses and the resistant genotypes were the pollen donors. Biparental crosses were advanced using the single-seed-descent method to generation F_6_, except for the biparental crosses involving the resistant genotypes PI 192051, PI 244061, and PI 195693, which were advanced to generation F_3_. The biparental populations were screened at the seedling stage with *P. triticina* race BBBQJ during winter months (December to February) in the biosafety level-two facility at the Agricultural Experiment Station Greenhouse Complex in Fargo, ND, in generations F_1_, F_2_, F_3_, and F_6_.

The single-pustule isolate CA1.2 of race BBBQJ was originally isolated from a sample collected from durum wheat fields in Cal-ifornia. Its virulence/avirulence phenotype was given based on infection types (IT) at the seedling stage on the international differential sets of ‘Thatcher’ wheat near-isogenic lines, with each line carrying a single *Lr* resistance gene (Long and Kolmer 1989). The virulence/ avirulence profile of race BBBQJ is *LrB*, *10*, *14b*, *20*, and *39*/*Lr1*, *2a*, *2c*, *3a*, *3ka*, *3bg*, *9*, *11*, *14a*, *16*, *17*, *18*, *24*, *26*, *28*, and *30*.

The inheritance of the gene or genes was determined in each of the biparental crosses. For the crosses that were evaluated at F_1_, five to six seeds were evaluated for response to race BBBQJ. For the crosses that were tested at the F_2_ stage, 118 to 342 plants were evaluated for disease response. At the F_3_ generation, approximately 18 to 30 seedlings from each F_3_ family (101 to 255 families/population) were screened. The F_6_ recombinant inbred lines (RIL) from each tested population were evaluated in a randomized complete block design, with three replications with five to eight seeds from each RIL per replicate. For all tests, the seedlings were grown in the greenhouse as described by Kertho et al. (2015). The resistant and susceptible parents of each cross, the susceptible durum wheat genotype ‘RL6089’, and the susceptible common wheat Thatcher were included in each tray as checks. Two replicates of differentials of Thatcher near-isogenic lines were planted alongside each experiment to confirm the purity of the race BBBQJ. Urediniospore increase, inoculation, incubation, and greenhouse conditions were completed as previously described by Aoun et al. (2016).

**TABLE 1:** Origin, type, and reaction to leaf rust and stem rust of the parental genotypes used in the crosses

Parents of the crosses	Type	Origin	IT to BBBQJ*^a^*	IT to TTKSK*^b^*
PI 534304	Landrace	Ethiopia	;1—	2
PI 192051	Landrace	Portugal	0;	2-
PI 313096	Landrace	Cyprus	;1—	.
PI 387263	Landrace	Ethiopia	;1	.
PI 209274	Breeding line	Australia	;1	.
PI 278379	Landrace	Malta	;1+	.
PI 244061	Landrace	Yemen	;1	.
PI 195693	Landrace	Ethiopia	;	.
Rusty^c^	Line	North Dakota	3+	3+
Divide^c^	Cultivar	North Dakota	3	.

a Infection types (IT) of the parental genotypes to *Puccinia triticina* race BBBQJ.

b IT of the parental genotypes to *P. graminis* f. sp. *tritici* race TTKSK.

c Susceptible parents of the crosses.

Leaf rust IT were assessed on the second-leaf stage 12 days after inoculation using a 0-to-4 scale (Long and Kolmer 1989; McIntosh et al. 1995), where IT 0 = no disease symptom, ; = hypersensitive flecks, 1 = small uredinia surrounded by necrosis, 2 = small-to medium-size uredinia surrounded by chlorosis, 3 = medium-size uredinia with no chlorosis or necrosis, and 4 = large uredinia with no chlorosis or ne-crosis. The mesothetic reaction (X reaction) is a mixture of fleck and higher infection types evenly distributed on the leaf surface. The seedlings showing IT of 0 to 2+ and X were considered resistant, whereas the plants showings IT of 3 and 4 were considered susceptible (Long and Kolmer 1989; McIntosh et al. 1995).

Based on the IT, the F_2_ plantswere classified as resistant (R) orsus-ceptible (S). The F_3_ families and the RIL were classified as homo-zygous resistant (HR), segregating (Seg), and homozygous susceptible (HS). The number of genes that were involved in the inheritance of leaf rust resistance were estimated based on segregation ratios and χ^2^ goodness-of-fit tests. The segregating F_6_-derived RIL were excluded when computing the *P* values of the χ^2^ test because only approximately 3% of the RIL were expected to be segregating.

**Characterization of stem rust resistance inheritance in two biparental crosses**. Two of the biparental populations that were described above, Rusty × PI 534304 and Rusty × PI 192051, were also screened with *P. graminis* f. sp. *tritici* race TTKSK (isolate 04KEN156/04) at the seedling stage at generation F_3_. The genotype PI 192051 was previously reported to be resistant to race TTKSK by Olivera et al. (2012), whereas PI 534304 was identified to be resistant to race TTKSK in the current study. Rusty was the susceptible parent to *P. graminis* f. sp. *tritici* race TTKSK. The avirulence/virulence profile of race TTKSK is *Sr24*, *36*, *Tmp/Sr5*, *6*, *7b*, *8a*, *9a*, *9b*, *9d*, *9e*, *9g*, *10*, *11*, *17*, *21*, *30*, *31*, *38*, *McN*.

The disease screenings were conducted in a biosafety level-three facility at the University of Minnesota, St. Paul. Twenty plants of each F_3_ family were inoculated approximately 10 days after planting with *P. graminis* f. sp. *tritici* race TTKSK. Urediniospores, stored at –80°C, were heat shocked at 45°C for 15 min, then rehydrated at room temperature under a relative humidity of 80% created with a KOH solution (Rowell 1984). The plants were inoculated as previ-ously described by Rouse et al. (2012). Thereafter, the plants were transferred to a greenhouse maintained at 18 ± 2°C with a 16-h photoperiod until evaluation of disease. Stem rust IT were assessed 14 days after inoculation using the 0-to-4 Stakman scale (Stakman et al. 1962). Seedlings showing IT of 0 to 2+ were considered resistant and those with IT of 3 to 4 were considered susceptible.

Based on the IT, the F_3_ families were classified as HR, Seg, or HS. The segregation ratios were analyzed using c^2^ goodness-of-fit tests. This allowed for the estimation of the number of genes involved in the inheritance of stem rust resistance. The number of families evaluated for Rusty × PI 534304 and Rusty × PI 192051 were 131 and 118, respectively.

BSA. Based on the inheritance study, four biparental populations that carry single *Lr* genes and one population that carries a single *Sr* gene were chosen for BSA. Leaf tissues from each population were collected from the F_2_ plants. This was done before the plants were advanced to the next generation.

The genomic regions associated with response to *P. triticina* race BBBQJ were identified in the biparental populations Divide × PI 313096, Rusty × PI 387263, Rusty × PI 209274, and Divide × PI 244061. For the populations derived from Rusty × PI 209274 and Divide × PI 244061, BSAwas performed using DNA extracted from 10 HR and 10 HS F_2_ plants. The homozygous F_2_ plants were identified by phenotyping F_2:3_ seedlings. For the remaining two populations, BSAwas done using DNA extracted from 20 to 22 HR and 20 to 22 HS F_6_ RIL.

The biparental cross Rusty × PI 534304 was used to locate the genomic region associated with response to *P. graminis* f. sp. *tritici* race TTKSK. The DNA of 16 HR and 16 HS RIL were used in the BSA. Because this population was screened with race TTKSK only at the F_3_ generation, The HR and HS F_6_ RIL were identified for BSA based on the phenotype of the corresponding F_2:3_ families.

The DNA of HR and HS plants was extracted using a cetyltrimethylammonium bromide extraction method described by Riede and Anderson (1996) and modified by Liu et al. (2006). Ad-ditional modifications of lyophilizing and grinding the leaf tissue were as described by Rouse et al. (2012). The DNA was then diluted to 50 ng/µl and pooled in equal volumes to obtain resistant andsusceptible bulks, as described by Michelmore et al. (1991). The HR and HS bulks and parents in each of the crosses were genotyped using Illumina’s custom wheat iSelect 9K SNP array (Cavanagh et al. 2013) at the USDA-ARS Small Grain Genotyping Laboratory in Fargo, ND. The data generated were scored using Illumina Genome Studio software.

**Response of the resistant genotypes to *P. triticina* races virulent to known *Lr* genes mapped in durum wheat cultivars**. In order to verify whether the resistant genotypes that were used to develop the biparental crosses carry previously characterized *Lr* genes in durum wheat cultivars, *P. triticina* races with virulence to *Lr3a, Lr14a*, *Lr27*+*31*, *Lr61*, and *Lr72* were used to phenotype the parents of the crosses.

Eleven durum cultivars were also included in this test, including ‘Alred’ as a susceptible check, the susceptible parents of the crosses (Rusty and Divide), ‘Llareta INIA’ carrying *Lr14a* (Herrera-Foessel et al. 2008a), ‘Camayo’ carrying *LrCamayo* (Herrera-Foessel et al. 2007), ‘Jupare C2001’ carrying *Lr27*+*31* (Singh and McIntosh 1984; Singh et al. 1993), ‘Guayacan INIA’ carrying *Lr61* (Herrera-Foessel et al. 2008b), ‘Capelli’, ‘Mindum’, ‘Russello’, and ‘Mexicali75’. The *P. triticina* races used were BBBSJ, CBBQS, and BBB/BN_*Lr61* vir. Race BBB/BN_*Lr61* vir is avirulent to *Lr72*, which is widely present in CIMMYT’s durum germplasm (Herrera-Foessel et al. 2014a) and virulent to *Lr10*, *Lr23,* and *Lr61*. The race BBBSJ was collected from durum in Spain in 2014 and is virulent to *LrB*, *Lr10*, *Lr14a*, *Lr14b*, *Lr20, Lr23*, and *Lr72*. The race CBBQS (also called CBG/BP based on the CIMMYT differential sets) was collected from durum fields in Mexico in 2008 and is virulent to *LrB*, *Lr3a*, *Lr3bg*, *Lr10, Lr14b*, *Lr23, Lr27*+*31*, and *Lr72* (Huerta-Espino et al. 2009) (J. Huerta-Espino, personal communication).

**Mapping of *Lr* genes in PI 209274, PI 387263, and PI 244061**. Based on the results of the BSA, we selected three bipa-rental populations (Rusty × PI 209274, Rusty × PI 387263, and Divide × PI 244061) to complete linkage mapping of the *Lr* trait and molecular markers. These populations were chosen because they were thought to carry previously uncharacterized *Lr* genes in durum cultivars. F_6_ RIL for populations Rusty × PI 209274 and Rusty × PI 387263 and F_2_ plants of the cross Divide × PI 244061 were used for linkage mapping.

In total, 130 RIL of the cross Rusty × PI 209274 and 97 RIL derived from Rusty × PI 387263 that were phenotyped using *P. triticina* race BBBQJ were genotyped with their respective markers identified during the BSA and additional markers from the 90K teteraploid consensus map (Maccaferri et al. 2015). In all, 11 SSR and 34 kompetitive allele-specific polymerase chain reaction (KASP) markers were used to genotype the susceptible parent (Rusty) and the resistant parent (PI 209274).

For the population Rusty × PI 387263, 23 KASP markers were used to genotype the susceptible parent (Rusty) and the resistant parent (PI 387263). Only the markers showing clear polymorphism between the parents were used to genotype the RIL.

For the population derived from Divide × PI 244061, 93 F_2_ plants were used for mapping. The HR, Seg, and HS F_2_ plants were identified based on the phenotype of the corresponding F_2:3_ families. Thirty-four KASP markers identified during BSA, with additional markers from the 90K tetraploid consensus map, were used to differentiate the susceptible parent (Divide) and the resistant parent (PI 244061). Thereafter, the polymorphic markers were used to screen the 93 F_2_ individuals derived from this cross. For the SSR markers that were used to genotype the parents and the RIL of the cross Rusty × PI 209274, the polymerase chain reactions (PCR) were accomplished in 25-µl volumes. Each reaction contained 1 µl of 10 µM forward primer, 1 µl of 10 µM reverse primer, 2.5 µl of 2.5 mM dNTP, 5 µl of 5× Green Go Taq Flexi buffer, 2.5 µl of 25 mM MgCl_2_, 0.15 µl of GoTaq Flexi DNA (Promega Corp.) at 5 U/µl, 10.85 µl of H_2_O, and 2 µl of DNA at 30 ng/µl. The PCR were performed in thermal cyclers programed to denature the DNA at 94°C for 5 min, followed by 35 cycles of 30 s of a 94°C denaturation step, 30 s of an annealing step (depending on the annealing temperatures of the respective SSR markers), and 45 s of a 72°C extension step. The program was then finished with a final 7-min extension step at 72°C and a 4°C permanent hold. The PCR products were separated on 3% agarose gels and DNA was visualized under UV light after staining with gel red nucleic acid gel stain (Biotium).

For the KASP markers, the primer sequences were obtained from the polymaker website (http://polymarker.tgac.ac.uk/). For each KASP marker, three primers were used in PCR. Two of them are allele-specific forward primers which result in biallelic discrimination and one common reverse primer (Ramirez-Gonzalez et al. 2014, 2015). Oligos, carrying standard FAM or HEX compatible tails (FAM tail: 59GAAGGTGACCAAGTTCATGCT39 or HEX tail: 59GAAGGTCG GAGTCAACGGATT39) were added to the forward primer sequences with the target SNP at the 39 end (Ramirez-Gonzalez et al. 2014). The PCR were in 10-µl volumes and prepared as described by the manufacturer (LGC). Each reaction contained 0.25 µl of 10 µM each forward primer, 0.5 µl of 10 µM reverse primer, 5 µl of KASP 2× master mix (LGC), 1 µl of H_2_O, and 3 µl of DNA at 30 ng/µl. PCR were placed in Multiplate 96-well unskirted PCR plates (MLP-9601; Bio-Rad) and sealed with an optical plate seal. The PCR were performed in a Bio-Rad CFX-96 real-time system thermal cycler programed as follows: hot-start activation at 94°C for 15 min followed by 10 touchdown cycles of denaturation at 94°C for 20 s and annealing or elongation (61 to 55°C) for 60 s, with a drop of 0.6°C per cycle. This was followed by 26 cycles of a denaturation step at 94°C for 20 s and an annealing or elongation step at 55°C for 60 s. The PCR plate was read at 37°C and fluorescent end-point genotyping was carried out. Data analysis was performed with the genotype cluster analysis software Bio-Rad CFX Manager 3.1 using the allelic discrimination option. If genotype clusters were not clearly defined after the initial KASP thermal cycle, the plate was thermally cycled for an additional three cycles of a denaturation step at 94°C for 20 s and an annealing or elongation step at 57°C for 60 s and the PCR plate was read again at 37°C. In some cases, the latter cycling and reading was repeated until distinct genotyping clusters were obtained.

For linkage mapping, the phenotypic responses were converted into binary data based on classification as resistant or susceptible IT. Then, the phenotypic and genotypic data were combined to gen-erate linkage maps using MapDisto.1.7.7.0.1.1 (Lorieux 2012), with minimum logarithm of odds (LODmin) = 7.0 and maximum recombination frequency of 0.3. The Kosambi mapping function was used to calculate genetic distance between markers (Kosambi 1943).

## Results

The inheritance of leaf rust resistance. The number of genes conferring resistance against *P. triticina* race BBBQJ in the eight durum wheat genotypes was determined by evaluating the IT at seedling stage of F_1_ plants and the segregation ratios of F_2_, F_3_, and F_6_ progenies (Table 2). In six of the crosses (Rusty × PI 192051, Divide × PI 244061, Rusty × PI 387263, Rusty × PI 209274, Rusty × PI 534304, and Divide × PI 313096), the F_1_ plants showed resistant IT to *P. triticina* race BBBQJ, suggesting that the resistance was dominant. The F_1_ plants of the cross Divide × PI 278379 were susceptible to BBBQJ, indicating that the resistance was recessive (Table 2).

Evaluation of 170 F_3_ families derived from the cross Rusty × PI 192051 showed a segregation ratio of 1:2:1 HR/Seg/HS (*P* = 0.33), suggesting that the *P. triticina* race BBBQJ resistance in PI 192051 is conferred by a single dominant gene. Similarly, evaluation of 255 F_3_ families and 98 F_6_ RIL of the cross Divide × PI 313096 segregated as 1:2:1 HR/Seg/HS (*P* = 0.06) and 1:1 HR/HS (*P* = 0.05), respectively which also fit the expected Mendelian ratios for a single gene. Therefore, the *Lr* gene in PI 313096 is conferred by a single dominant gene (Table 2).

The segregation ratios of 311 F_2_ plants generated from the cross Divide × PI 244061 fit 3:1 R/S (*P* = 0.77). Further screening of 117 F_3_ families of the same cross showed a segregation of 1:2:1 HR/Seg/HS (*P* = 0.06) which suggests that a single dominant resistance gene confers resistance to *P. triticina* race, BBBQJ in PI 244061 (Table 2).

In the cross of Rusty × PI 387263, the 106 F_3_ families and 140 RIL evaluated segregated as 1:2:1 HR/Seg/HS (*P* = 0.16) and 1:1 HR/HS (*P* = 0.10), respectively. This indicated that a single dominant resistance gene controls the resistance to *P. triticina* race BBBQJ in PI 387263 (Table 2).

**Table 2:** Characterization of leaf rust resistance (*Puccinia triticina* race BBBQJ) inheritance at the seedling stage in eight resistant durum genotypes based on infection types of F_1_ plants and segregation ratios at F_2_, F_3_, and F_6_^a^

Populations			F_1_				F_2_ segregation ratios	F_3_				segregation ratios	F_6_			segregation ratios
			R/S^b^			*P* for χ2	HR/Seg/HS^c^				*P* for χ2	HR/Seg/HS^d^			HR/HS	*P* for χ2
			Segregation(*n*)		Expectedratio		Segregation(n)			Expectedratio		Segregation(*n*)			Expectedratio	
Rusty x PI 534304		1+	^-^		^-^	^-^	17:79:33			1:2:1/1:8:7	0.005*/1.7E-05*	114:2:61			1:1-3:1	<1E-05*/0.03*
Rusty x PI 192051		;1	^-^		^-^	^-^	37:89:44			1:2:1	0.33	-			-	-
Divide x PI 313096		1+	^-^		^-^	^-^	62:144:49			1:2:1	0.06	57:3:38			1:1	0.05
Rusty x PI387263		1+	^-^		^-^	^-^	18:58:30			1:2:1	0.16	76:7:57			1:1	0.10
Rusty x PI 209274		^1^_-_^+^	253:89		3:1	0.66	39:78:37			1:2:1	0.78	62:8:60			1:1	0.86
Rusty x PI 278379			47:166		1:3/3:13^e^	0.32/0.22	4:48:50			1:8:7	0.43	22_:_6:65			1:3	0.___95
Divide x PI 278379		3	31:172		3:13	0.20	^-^			^-^	^-^	-			-	-
Divide x PI 244061		^1^_-_^+^	231:80		3:1	0.77	19:69:29			1:2:1	0.06	-			-	-
Rusty x PI 195693			36:82		1:3	0.38	^-^			^-^	^-^	-			-	-
Divide x PI 195693		^-^	88:125		7:9	0.48	18:52:31			1:8:7	0.18	-			-	-

a Symbols:- indicates population was not evaluated at this generation and

* indicates *P* value where the observed segregation ratio is significantly different from the expected segregation ratio at a 95% level of confidence.

b Number of resistant (R) and susceptible (S) F_2_ progenies.

c Number of homozygous resistant (HR), segregating (Seg), and homozygous susceptible (HS) F_3_ families.

d Number of homozygous resistant (HR), segregating (Seg), and homozygous susceptible (HS) recombinant inbred lines at F_6_ generation.

e Observed segregation ratios could fit into two possible expected segregation ratios (1:3 R/S or 3:13 R/S).

The F_2_ population (342 plants) of the cross Rusty × PI 209274 segregated as 3:1 R/S (*P* = 0.66) whereas the segregation ratio of 154 F_3_ families was 1:2:1 HR/Seg/HS (*P* = 0.78) and the F_6_ RIL segregated as 1:1 HR/HS (*P* = 0.86). This suggests that a single dominant gene conferred the observed resistance in PI 209274 (Table 2).

All five F_1_ plants derived from the cross Rusty × PI 534304 showed resistant IT, indicating that the resistance to *P. triticina* race BBBQJ is dominant. The subsequent screening of 129 F_3_ and 177 F_6_ RIL resulted in segregation of 17:79:33 H/Seg/HS and 144:2:61 HR/Seg/HS, respectively, which did not fit Mendelian inheritance for one or two genes, based on *P* values of the c^2^ test (<0.05) at a 95% level of confidence (Table 2).

The segregation pattern of cross Rusty × PI 278379 showed that F_2_ segregation ratios could fit two possible models. One of the models was 1:3 R/S (*P* = 0.32), which suggests the presence of a single recessive gene controlling resistance to *P. triticina* race BBBQJ. The observed segregation at F_2_ also fit a 3:13 R/S ratio (*P* = 0.22), which indicates the involvement of two genes: one dominant gene suppressing the expression of another dominant resistance gene. The same segregation ratio (3:13 R/S; *P* = 0.20) was obtained by crossing the same resistant parent PI 278379 with the susceptible parent Divide. Further evaluation of the population Rusty × PI 278379 showed fit to two ratios: 1:8:7 HR/Seg/HS (*P* = 0.43) and 1:3 HR/HS (*P* = 0.95) for F_3_ families and F_6_ RIL, respectively. These results suggest that two genes are most likely involved in this population (Table 2).

Two populations were developed for the resistant genotype PI 195693. Evaluation of each population suggested different modes of inheritance. The segregation ratio of 118 F_2_ plants of the cross Rusty × PI 195693 fit 1:3 R/S(*P* = 0.38), indicating thatthe resistance was conferred by a single recessive gene. However, the F_2_ plants (213 individuals) of the cross Divide × PI 195693 segregated as 7:9 R/S (*P* = 0.48), indicating the presence of two recessive genes. Further screening of the F_3_ lines of Divide × PI 195693 were distributed in accordance with a 1:8:7 HR/Seg/HS ratio, indicating the presence of two genes (Table 2).

Stem rust resistance inheritance. The inheritance of stem rust resistance to *P. graminis* f. sp. *tritici* race TTKSK in the two pop-ulations Rusty × PI 534304 and Rusty × PI 192051 was determined based on the evaluation of F_3_ progenies.

**Table 3:** Characterization of stem rust resistance (*Puccinia graminis* f. sp. *tritici* race TTKSK) inheritance at seedling stage in two resistant durum lines based on segregation ratios of F_3_ progenies^a^

Characterization	Rusty × PI 534304	Rusty × PI 192051
Homozygote resistant	27	31
Segregating	69	70
Homozygote susceptible	35	17
Expected segregation ratio	1:2:1 HR/Seg/HS	1:2:1 HR/Seg/HS
*P* value of χ^2^	0.51	0.02*

a HR = homozygous resistant, Seg = segregating, and HS = homozygous susceptible. An asterisk

(*) indicates *P* value where the observed segregation ratio is significantly different from the expected segregation ratio at a 95% level of confidence

The 131 F_3_ families of the biparental cross Rusty × PI 534304 fit 1:2:1 HR/Seg/HS (*P* = 0.51), which suggested that PI 534304 carriesasingle *Sr* genecontrollingtheresistanceto TTKSK. The segregation observed in the cross Rusty × PI 192051 was 31:70:17 HR/Seg/HS, which did not fit segregation for a single gene based on the *P* value of the c^2^ test (*P* = 0.02) (Table 3).

**BSA**. Genomic regions associated with *Lr* and *Sr* genes were identified via BSA in five biparental populations in which the resistance appeared to be conferred by single dominant resistance genes. Four of these crosses were used to map the chromosomal regions associated with *Lr* resistance to *P. triticina* race BBBQJ, whereas one cross was used to identify the region associated with the *Sr* gene conferring resistance to *P. graminis* f. sp. *tritici* race TTKSK (Table 4).

*Divide* × PI 244061 *population.* Thirty-three SNP located on chromosome 2B were associated with leaf rust response in the cross involving Divide × PI 244061. The positions of the SNP markers were based on the hexaploid consensus map (Cavanagh et al. 2013). Based on the BLASTn of the SNP sequences against the Chinese Spring chromosome survey sequences (https://urgi.versailles.inra. fr/blast/?dbgroup=wheat_all&program=blastn), six markers were found on chromosome arm 2BL, while the rest of the markers were on 2BS (Table 4; Supplementary Table S1).

*Rusty* × *PI 209274 and Divide* × *PI 313096 populations.* The leaf rust resistance in the cross Rusty × PI 209274 was associated with 10 SNP on chromosome arm 6BS (Table 4).

Six SNP on chromosome arm 6BS were associated with leaf rust response in the population Divide × PI 313096. Even though the *Lr* genes in PI 209274 and PI 313096 were both located on 6BS, the BSA did not reveal any common SNP linked with response to *P. triticina* race BBBQJ between the two populations. However, the majority of the trait-associated SNP in both populations mapped to overlapping regions between 0.6 and 14.5 centimorgans (cM), based on the hexaploid consensus map of Cavanagh et al. (2013) (Table 4).

*Rusty* × *PI 387263 population.* Five SNP associated with leaf rust response were detected on chromosome arm 6BL in the cross Rusty × PI 387263.

*Rusty* × *PI 534304 population.* Thirty-two SNP on chromosome arm 6AL were associated with stem rust response to race *P. graminis* f. sp. *tritici* race TTKSK in the cross Rusty × PI 534304 (Table 4).

**Response of the parental genotypes to *P. triticina* races virulent to known *Lr* genes in durum**. The parents resistant to *P. triticina* race BBBQJ that were used to develop the biparental populations alongside other durum cultivars were screened using *P. triticina* races BBBSJ, CBBQS, and BBB/BN_*Lr61*vir. The IT indicated that race BBBSJ, which carries virulence to *LrB*, *Lr10*, *Lr14a*, *Lr14b, Lr23, Lr20,* and *Lr72*, was avirulent to all the resistant parents of the crosses and on Camayo and Juapare C2001 durum wheat. Race CBBQS, virulent to *LrB*, *Lr3a*, *Lr3bg*, *Lr10*, *Lr14b*, *Lr23*, *Lr27*+*31*, and *Lr72*, was avirulent to the eight resistant pa-rental genotypes used in the crosses and to Camayo and Llareta INIA. Race BBB/BN_*Lr61* vir, which carries virulence on *Lr10*, *Lr23*, and *Lr61*, was avirulent to all the eight genotypes and cultivars, except PI 313096, Alred, and Guayacan INIA. This suggests that the resistance in the eight genotypes used to develop the biparental populations is conferred by different or additional genes than the previously characterized *Lr* genes in durum cultivars, including *Lr3a*, *Lr14a*, *Lr27*+*31*, *Lr61,* and *Lr72*, except PI 313096, which most likely carries *Lr61* (Table 5).

**Table 4:** Generation, trait, number of plants in homozygous resistant (HR) and homozygous susceptible (HS) bulks of the biparental crosses used in the bulked segregant analysis (BSA), and results of BSA

Populations	Generation	Trait	Pathogen race	HS bulk (*n*)^a^	HR bulk (*n*)^b^	Chromosome	SNP (*n*)^c^	Possible gene
Divide × PI 313096	F_6_	Leaf rust	BBBQJ	20	20	6BS	6	*Lr61*
Rusty × PI 387263	F_6_	Leaf rust	BBBQJ	22	22	6BL	5	Possibly novel
Rusty × PI 209274	F_2_	Leaf rust	BBBQJ	10	10	6BS	10	*Lr53* or possibly novel
Divide × PI 244061	F_2_	Leaf rust	BBBQJ	10	10	2B	33	*Lr13* or possibly novel
Rusty × PI 534304	F_6_	Stem rust	TTKSK	16	16	6AL	32	*Sr13*

a Number of HS F_2_ plants or recombinant inbred lines (RIL) included in the HS bulk.

b Number of HR F_2_ plants or RIL included in the HR bulk.

c Number of associated single-nucleotide polymorphisms (SNP) with rust response. Markers linked with rust response in these populations are presented in Supplementary Table S1.

**Mapping of the *Lr* gene in PI 209274**. The population Rusty × PI 209274 was selected for linkage mapping using 130 F_6_ RIL. The identified SNP on 6BS that were associated with leaf rust response in this population using BSA spanned a genomic region of 21.9 cM, based on the consensus map of Cavanagh et al. (2013) (Table 4).

**Fig. 1:**
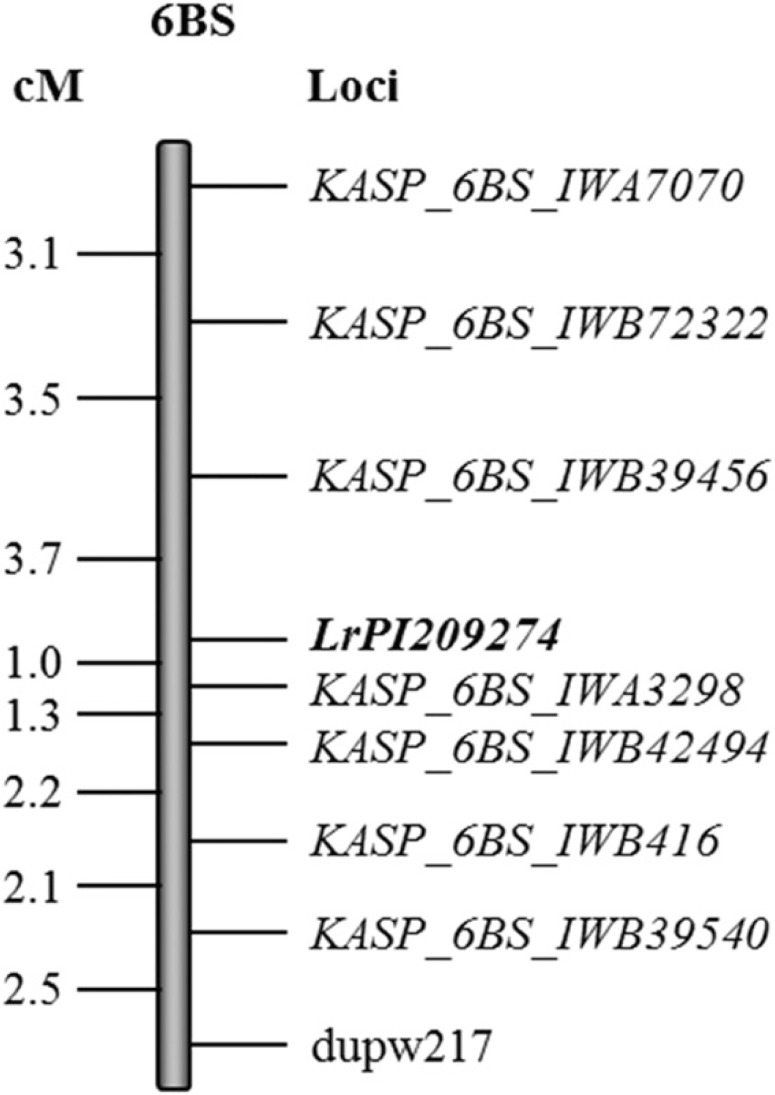
Distance in centimorgans (cM) between simple sequence repeat (SSR) and kompetitive allele-specific polymerase chain reaction (KASP) assay single-nucleotide polymorphism markers linked to the leaf rust (*Puccinia triticina* race BBBQJ) resistance gene (*LrPI209274*) on chromosome arm 6BS using phenotypic and genotypic data of the recombinant inbred lines of the cross Rusty × PI 209274 at F_6_ generation.

The SNP markers identified in the biparental cross Rusty × PI 209274 using BSAwere used to develop KASP markers, asdescribed by Ramirez-Gonzalez et al. (2014). Three KASP markers (*KASP_6BS_IWA7070*, *KASP_6BS_IWA3298*, and *KASP_6BS_IWA4290*) gave clear poly-morphism between the resistant parent (PI 209274) and the suscep-tible parent (Rusty). Therefore, these KASP markers were used initially to genotype the RIL of this biparental population. The mapping of the *Lr* gene associated with leaf rust response to *P. triticina* race BBBQJ in PI 209274 showed that the gene was initially flanked by *KASP_6BS_IWA3298* and *KASP_6BS_IWA7070*. Therefore, ad-ditional SNP and SSR markers located between these two markers, based on the tetraploid consensus map (Maccaferri et al. 2015), were used to genotype the parents of the cross. Five KASP assay SNP and one SSR (*dupw217*) markers that were polymorphic between the parents were then used to genotype the F_6_ RIL. The mapping identified two flanking markers (*KASP_6BS_IWA3298* and *KASP_6BS_IWB39456*) that delineated the *Lr* gene resistant to race BBBQJ, here designated as *LrPI209274* (Fig. 1).

The distance between the flanking markers was 4.7 cM. The marker *KASP_6BS_IWA3298* was the most closely linked to *LrPI209274* at a distance of 1.0 cM whereas *KASP_6BS_IWB39456* was located at 3.7 cM distal to *LrPI209274.* The rest of markers were located further away from the gene, with most of them distal to the gene (Fig. 1). All linked markers with *LrPI209274* (Fig. 1) in this durum population conformed to the expected ratio of 1:1 at a 95% level of confidence (*P* values of χ^2^ tests ranged from 0.13 to 0.84 for the KASP markers and *P* = 0.05 for the SSR marker *dupw217*). The primer sequences of the KASP markers used for mapping of *LrPI209274* as well as the alleles associated with resistance are presented in Table 6.

**Fig. 2:**
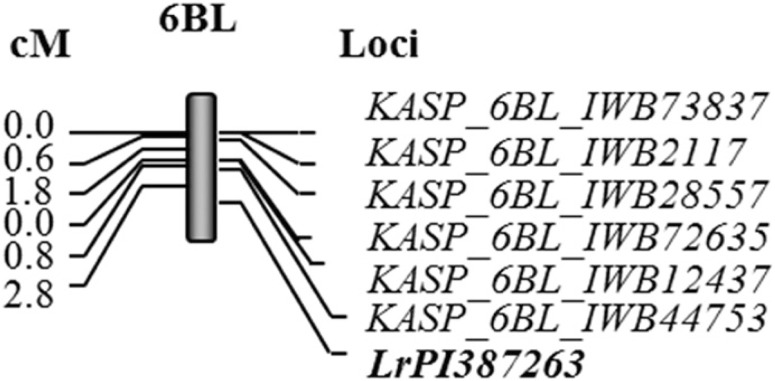
Distance in centimorgans (cM) between kompetitive allele-specific polymerase chain reaction (KASP) assay single-nucleotide polymorphism markers linked to the leaf rust (*Puccinia triticina* race BBBQJ) resistance gene (*LrPI387263*) on chromosome arm 6BL using phenotypic and genotypic data of the recombinant inbred lines of the cross Rusty × PI 387263 at F_6_ generation.

Mapping of the *Lr* gene in PI 387263. For the population Rusty × PI 387263, the BSA revealed five SNP on 6BL that are associated with leaf rust response. Based on the 9K wheat consensus map (Cavanagh et al. 2013), these markers span a genomic region of 30.0 cM. Because the number of SNP identified during the BSAwas limited, additional SNP from the 90K tetraploid consensus map were used for further genotyping to saturate the region. The SNP from the BSA and others falling within the regions were used to develop KASP markers for further testing. All the polymorphic markers between the two parents were subsequently applied to screen the RIL. Initial mapping showed that the *Lr* gene associated with leaf rust response to *P. triticina* race BBBQJ in PI 387263 is located distal to *KASP_6BL_IWB72635.* Additional KASP markers found distal to marker *KASP_6BL_IWB72635* were used to more accurately map the *Lr* gene. The final mapping showed that *KASP_6BL_IWB44753* was the closest and mapped at a distance of 2.8 cM from the gene. The *Lr* gene in PI 387263 mapped at the distal end of chromosome 6BL and is hereby designated as *LrPI387263* (Fig. 2).

**Table 5:** Infection types of the parental genotypes of the crosses and durum wheat cultivars to *Puccinia triticina* races BBBSJ, CBBQS, and BBB/BN_*Lr61*vir at the seedling stage

Entries	BBBSJ^a^	CBBQS^b^	BBB/BN_*Lr61*vir^c^
PI 534304	0;	;	;1–
PI 192051	;	;1	;
PI 313096	0;	0;	3+
PI 387263	;	;1	;1–
PI 209274	;1+	…	X
PI 278379	;2+ C	2+C	;1+
PI 244061	;	;	…
PI 195693	;	;1	;1
Rusty	3	3	…
Divide	2+3	3	…
Alred	3	4	3+
Llareta INIA	3	;13–	X
Camayo	;1–	;1	;1
Jupare C 2001	;1	3	;1
Capelli	2+3	3+	…
Mindum	3	3	…
Russello	3	3	…
Mexicali 75	3	3	…
Guayacan INIA	…	…	3

a P. triticina race virulent to LrB, Lr10, Lr14a, Lr14b, Lr20, Lr23, and Lr72.

b P. triticina race virulent to LrB, Lr3a, Lr3bg, Lr10, Lr14b, Lr23, Lr27+31, and Lr72.

c P. triticina race virulent to Lr10, Lr23, and Lr61.

KASP markers used for mapping *LrPI387263* deviated from the expected ratio of 1:1 at a 95% level of confidence based on the *P* values of χ^2^ tests, except *KASP_6BL_44753.* The primer sequences of the KASP markers used for mapping of *LrPI387263* as well as the alleles associated with resistance are presented in Table 6.

**Fig. 3:**
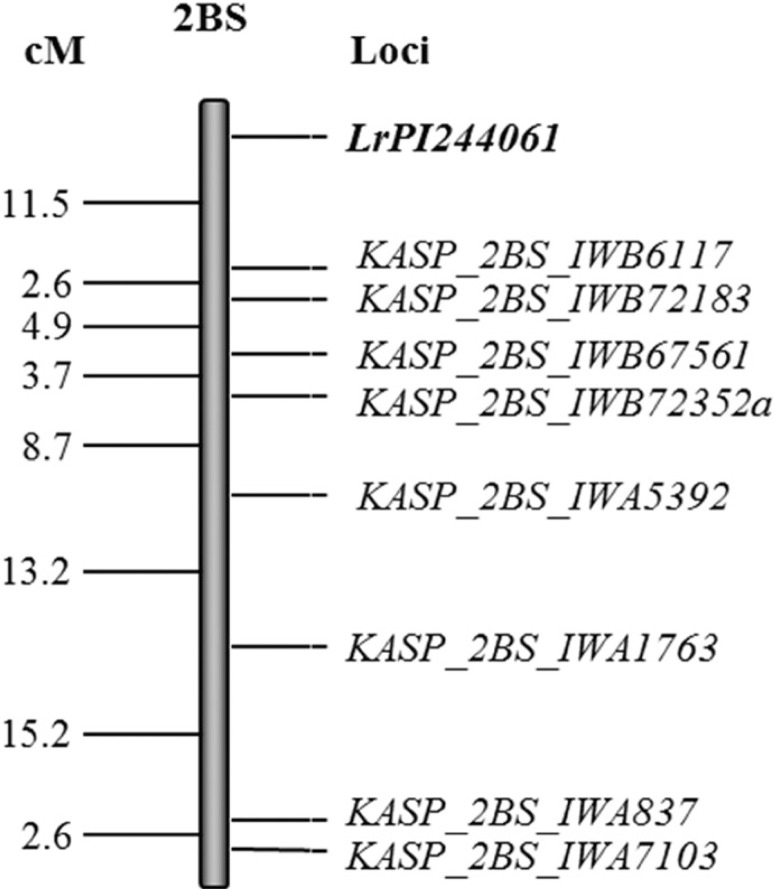
Distance in centimorgans (cM) between kompetitive allele-specific polymerase chain reaction (KASP) assay single-nucleotide polymorphism markers linked to the leaf rust (*Puccinia triticina* race BBBQJ) resistance gene (*Lr244061*) on chromosome arm 2BS using phenotypic and genotypic data of F_2_ plants of the cross Divide × PI 244061.

Mapping of the *Lr* gene in PI 244061. For the population Divide × PI 244061, the BSA revealed 33 SNP on chromosome 2B that are associated with leaf rust response. Based on the 9K wheat consensus map (Cavanagh et al. 2013), these markers occupy a genomic region spanning 98.6 cM. The polymorphic KASP markers, derived from the identified SNP during the BSA, were used to genotype the F_2_ progenies. Initial mapping indicated that the *Lr* gene in PI 244061 is located distal to *KASP_2BS_IWA5392.* Subsequently, more KASP markers found distal to *KASP_2BS_IWA5392* based on the 90K tetraploid consensus map were developed. Application of the polymorphic markers on F_2_ progenies mapped the *Lr* gene distal to *KASP_2BS_IWB6117* at a distance of 11.5 cM. This gene was hereby designated as *LrPI244061* (Fig. 3).

KASP markers used for mapping *LrPI244061* conformed to the expected ratio of 1:1 at a 95% level of confidence based on χ^2^ tests, except for *KASP_2BS_IWB67561*, *KASP_2BS_IWA5392*, *KASP_2BS_IWA1763*, and *KASP_2BS_IWA837*. The primer sequences of the KASP markers used for mapping of *LrPI244061* as well as the alleles associated with resistance are shown in Table 6.

### Discussion

*P. triticina* race BBBQJ, highly virulent on tetraploid wheat, was recently found in the southern Great Plains region of the United States (Kolmer 2015). Therefore, the spread of this race to North Dakota, the major durum-producing region of the United States, is possible. Because most of the North Dakota durum cultivars are susceptible to this race and few effective *Lr* genes are available to the durum wheat breeding programs globally, we aimed to identify new *Lr* genes. In the present study, resistance to *P. triticina* race BBBQJ was conferred by single dominant genes in five of the durum populations. BSA showed that the genomic locations of the *Lr* genes in in PI 209274, PI 244061, PI387263, and PI 313096 mapped to chromosome arms 6BS, 2BS, 6BL, and 6BS, respectively. This was a fast and relatively inexpensive method to identify that the resistance in these four genotypes was conferred by at least three different genes. This method assessed the genetic diversity of resistance in these genotypes and identified possible new *Lr* genes that can be used to broaden the genetic diversity of leaf rust resistance in durum wheat. Apart from being resistant to BBBQJ, the eight genotypes used to develop these populations showed a broad spectrum of resistance to several *P. triticina* races collected worldwide at the seedling stage in the greenhouse and at the adult-plant stage in field trials (Aoun et al. 2016). In addition, based on our results from the current study, these genotypes are resistant to *P. triticina* races virulent to commonly used *Lr* genes in durum breeding programs, including *Lr3a*, *Lr14a*, *Lr27*+*31*, *Lr61*, and *L72*, suggesting that new or underutilized *Lr* genes may be present in theses geno-types. The genotypes utilized were collected from different countries and seven of eight were landraces. Wheat landraces are known to carry new resistance genes to several diseases, including rust, because the use of landraces in the modern breeding programs is not frequent (Bonman et al. 2007; Bux et al. 2012; Gurung et al. 2014; Newton et al. 2010; Reif et al. 2005).

Our study showed that the *Lr* gene in PI 244061 was mapped to chromosome 2BS. Several previously mapped *Lr* genes on 2BS have been reported, including *Lr23* (McIntosh and Dyck 1975; Nelson et al. 1997; Watson and Luig 1961). However, PI 244061 was resistant to races BBBSJ and CBBQS, which are virulent to *Lr23*. Virulence to *Lr23* is common in *P. triticina* races isolated from durum wheat (Huerta-Espino and Roelfs 1992; Ordon˜ez and Kolmer 2007a; Singh et al. 2005). In addition, the map position of *LrPI244061* is distal to *Lr23*, which is tightly linked to *KASP_69462* (Chhetri et al. 2017). Other genes on 2BS include *Lr13* (Singh et al. 1992) and *Lr16* (Zhang and Knott 1990) that have been postulated in durum. *Lr16* is tightly linked to SSR (*wmc764* and *wmc661*) and SNP markers that are on the distal end of chromosome 2BS (Kassa et al. 2017; McCartney et al. 2005) However, the map position of *LrPI244061* is proximal to *Lr16*, based on the tetraploid consensus map (Maccaferri et al. 2015). Therefore, the *Lr* gene in PI 244061 is unlikely tobe *Lr16.* Because the *Lr13* Thatcherlineisresistantto *P. triticina* race BBBQJ at the seedling stage (Kolmer 2015), the *Lr* gene in PI 244061 is possibly *Lr13*. Another seedling resistance gene on 2BS, designated as *Lr73,* was mapped in the common wheat line ‘Morocco’ (Park et al. 2014). However, Morocco is highly susceptible to race BBBQJ, suggesting that *Lr73* is not the gene of interest in PI 244061. Therefore, the *Lr* gene in PI 244061 is possibly *Lr13* or a new *Lr* gene.

**Table 6:** Primers of kompetitive allele-specific polymerase chain reaction (KASP) assay markers derived from the 90 K iSelect assay for mapping leaf rust resistance genes effective to *Puccinia triticina* race BBBQJ in the populations Rusty × PI209274, Rusty × PI387263, and Divide × PI244061

Markers for mapping	Allele1 primer sequence^a^	Allele2 primer sequence^a^	Reverse primer sequence
LrPI209274			
KASP_6BS_IWA7070	accagtcgcagtggggtT	accagtcgcagtggggtC	aggagctgttgatgggcc
KASP_6BS_IWB72322	ttgaactcgtcggcgccT	ttgaactcgtcggcgccG	gcatgctacaccgagacaag
KASP_6BS_IWB39456	cttcggagcgtgctacaaT	cttcggagcgtgctacaaC	acaaacaaatgcagagcagtac
KASP_6BS_IWA3298	gcgtttgctcttgctgcA	gcgtttgctcttgctgcG	agtggttctagatttgggttca
KASP_6BS_IWB42494	agcttcggggtcaacttactA	agcttcggggtcaacttactG	aaaatctctacgctggatgagt
KASP_6BS_IWB416	tgggagaaacattagcatatgcaT	tgggagaaacattagcatatgcaC	tctactgatcatcatcatcgtgg
KASP_6BS_IWB39540	tcccattgtgttattttgtaagggT	tcccattgtgttattttgtaagggC	acgctgaaaccagggagttt
LrPI387263			
KASP_6BL_IWB73837	acctcttctttgtctcggcT	acctcttctttgtctcggcC	agaataagaacggccccgg
KASP_6BL_IWB2117	cgacatatccgtttgtcttgtcA	cgacatatccgtttgtcttgtcG	gtcattgactgccacggtca
KASP_6BL_IWB28557	gtagtgtctgtctttggcgT	gtagtgtctgtctttggcgC	gatgaagctgaccacttgcta
KASP_6BL_IWB72635	ggaatcatgtactcctgtacctT	ggaatcatgtactcctgtacctC	atatccggccgccactga
KASP_6BL_IWB12437	acgtgttcgggaatacagtgaA	acgtgttcgggaatacagtgaG	cacgcaaatgcctgaactcc
KASP_6BL_IWB44753	aggttgggatgaggctctcA	aggttgggatgaggctctcG	cgggttggagtctgacgatt
LrPI244061			
KASP_2BS_IWB6117	gatgtggtggaaccccaaT	gatgtggtggaaccccaaC	cgaaaaatgttagccgtctgattc
KASP_2BS_IWB72183	ctactaccaaactgacccaaaactT	ctactaccaaactgacccaaaactC	aatcggatgtgtgtgcacca
KASP_2BS_IWB67561	cgccgtaacctccctgttT	cgccgtaacctccctgttC	gaagtgaggaggaagccgag
KASP_2BS_IWB72352a	cacgggtaaatctgggaaaacT	cacgggtaaatctgggaaaacC	gagtgcagtttggcaacgag
KASP_2BS_IWA5392	tctaggaataaaagcaagagcacA	tctaggaataaaagcaagagcacG	agaacatcgcccgtagtgg
KASP_2BS_IWA1763	gacttacaagtgagcttctatgcT	gacttacaagtgagcttctatgcC	cgagctagcctgccgtgt
KASP_2BS_IWA837	atcgggttcgggctgatT	atcgggttcgggctgatC	gagaagaagagccccgtcaa
KASP_2BS_IWA7103	agtaatgtgtatcagtgccatcA	agtaatgtgtatcagtgccatcG	gtgtaccctgcagtcattcg

a Single-nucleotide polymorphism alleles: Allele 1 = HEX seq GAAGGTCGGAGTCAACGGATT and Allele2 = FAM seq GAAGGTGACCAAGTTCATGCT. Nucleotides in bold and uppercase are associated with the resistance.

The *Lr* genes in PI 209274 and PI 313096 were both located on chromosome arm 6BS. *Lr61* is the only known gene on 6BS in durum cultivars identified to date and was previously mapped in CIMMYT Guayacan INIA wheat (Herrera-Foessel et al. 2008a). The genotype PI 313096 was susceptible to *P. triticina* race BBB/BN_*Lr61*vir, suggesting that the resistance in PI 313096 is most likely *Lr61*. The latter is effective against the *P. triticina* race used in this study (BBBQJ). PI 209274 was resistant to BBB/BN_*Lr61*vir, indicating that the single dominant *Lr* gene in PI 209274 differs from *Lr61.*

Other *Lr* genes mapped on 6BS in wheat include *Lr36* originating from *Aegilops speltoides* (Dvoˇra´k and Knott 1990), *Lr53* from *T. turgidum* subsp. *dicoccoides* (Dadkhodaie et al. 2011; Marais et al. 2005), and *Lr59* originating from *A. peregrina* (Marais et al. 2008; Pirseyedi et al. 2015). The genes *Lr36* and *Lr59* were transferred to hexaploid wheat from wild relatives, which makes them unlikely to be the *Lr* gene in PI 209274. Therefore, the *Lr* gene in PI209274 is likely *Lr53* or a previously uncharacterized gene.

The *Lr* genes in the population Rusty X PI 387263 were located on chromosome arm 6BL. Herrera-Foessel et al. (2007) identified two linked genes in repulsion on chromosome 6BL that were effective against *P. triticina* race BBG/BN collected in Mexico: *Lr3a* and *LrCamayo*. The gene *Lr3a* that cosegregated with *Xmwg798* (Sacco et al. 1998) was confirmed to be present in ‘Storlom’ durum wheat (Herrera-Foessel et al. 2007). In the present study, PI 387263 is resistant to the *P. triticina* race CBBQS which is virulent to *Lr3a*, indicating that the resistance gene in PI 387263 is different from *Lr3a*. Further screening of Camayo and PI 387263 with *P. triticina* isolate Eth-63-1 (race EEEEE, avirulent on Thatcher) collected from durum wheat in Ethiopia showed virulence on PI 387263 but not on Camayo (M. Aoun, unpublished). This suggests that the resistance in PI 387263 is possibly conferred by a different gene from *LrCamayo*. Therefore, the *Lr* gene in PI387263 is likely new.

The genotype PI 195693 showed resistance to BBBSJ, CBBQS, and BBB/BN_*Lr61*vir. Therefore, the resistance in PI 195693 is conferred by a different gene or a gene in addition to *LrB*, *Lr3a*, *Lr3bg*, *Lr10, Lr14a, Lr14b*, *Lr20*, *Lr23, Lr27*+*31*, *Lr61*, and *Lr72.* The F_2_ segregation ratio of 1:3 R/S in the cross Rusty × PI 195693 (one recessive gene) and 7:9 R/S in the cross Divide × PI 195693 (two recessive genes) could be due to the difference in the genetic background of the susceptible parents Divide and Rusty. Even though the segregation ratio of 1:8:7 HR/Seg/HS at generation F_3_ in Divide × PI 195693 could confirm the presence of two recessive genes, the same ratio could also suggest the involvement of two complementary dom-inant genes. Similar segregation patterns at the seedling stage (sus-ceptible F_1_, 7:9 R/S at F_2_, and 1:8:7 HR/Seg/HS at F_3_) were observed previously in the cross ‘Atil C200’ × ‘Hualita’ to the Mexican *P. triticina* race BBG/BN (Herrera-Foessel et al. 2005). However, Herrera-Foessel et al. (2005) reported that the resistance in the cross Atil C200 × Hualita was due to the presence of two dominant complementary genes rather than two recessive genes because the F_1_ plants were resistant in the field. Only one single case of com-plementary genes with dominant interaction conditioning leaf rust resistance has been reported in durum wheat. Jupare C2001 and ‘Banamichi C2004’ durum wheat carry the complementary genes *Lr27*+*31* on chromosome arms 3BS and 4BL, respectively (Herrera-Foessel et al. 2005, 2014b) that were originally characterized in common wheat (Singh and McIntosh 1984; Singh et al. 1993).

The F_1_ plants of the cross Divide × PI 278379 were susceptible to *P. triticina* race BBBQJ, indicating the presence of recessive resistance (dominant susceptibility) to leaf rust. The segregation of 3:13 R/S in generation F_2_ of Rusty × PI 278379 and Divide × PI 278379 populations and the distribution of 1:8:7 HR/Seg/HS in the F_3_ families and 1:3 HR/HS in the F_6_ RIL of Rusty × PI 278379 could mean the involvement of one dominant resistance gene with one suppressor gene. A possible scenario for this ratio might be due to the presence of a dominant resistance gene in PI 278379 that is suppressed by a suppressor gene from the susceptible parent (Rusty or Divide). Cases of suppressor genes of rust resistance have been reported in wheat–rust pathosystems. For instance, a suppressor gene of *Lr23* designated as *SuLr23* on chromosome arm 2DS that was derived from *A. tauschii* was identified in synthetic hexaploid wheat (Nelson et al. 1997). In addition, suppressors of *Lr* genes have been identified in the A and B genomes in durum wheat (Assefa and Fehrman 2000). Knott (2000) also characterized suppressors of *Sr* genes in the A and B genomes in ‘Medea’ durum wheat.

The resistance to *P. triticina* race BBBQJ in the population Rusty × PI 534304 is dominant, whereas the segregation ratios at generations F_3_ and F_6_ did not fit expected segregation ratios for one or two genes. The same population was used to map an *Sr* gene effective to *P. graminis* f. sp. *tritici* race TTKSK. The resistance to race TTKSK in PI 534304 is conferred by a single resistance gene that is located on chromosome arm 6AL. Chromosome arm 6AL is also known to carry *Sr13* (Jin et al. 2007; Klindworth et al. 2007), which is commonly found in durum wheat cultivars. However, a diagnostic marker of *Sr13* is currently not available. The IT of PI 534304 and the segre-gating population to *P. graminis* f. sp. *tritici* race TTKSK were similar to that of *Sr13.* Therefore, the *Sr* resistance in PI 534304 is most probably *Sr13*. Unfortunately, this *Sr* gene is not effective against the select *P. graminis* f. sp. *tritici* races in Ethiopia such as JRCQC (Olivera et al. 2012).

PI 192051 carries a single dominant *Lr* gene effective to *P. triticina* race BBBQJ. Interestingly, PI 192051 showed a broad spectrum of resistance to several *P. triticina* races tested in a previous study by Aoun et al. (2016). In addition, PI 192051 is resistant to *P. graminis* f. sp. *tritici* race TTKSK. The stem rust resistance in the cross Rusty × PI 192051 did not follow the segregation ratio of a single gene. PI 192051 was not only resistant to *P. graminis* f. sp. *tritici* race TTKSK but also to *P. graminis* f. sp. *tritici* race JRCQC, with virulence to *Sr13* and *Sr9e*, which are common in durum wheat cultivars (Olivera et al. 2012). The genotype PI 192051 was also highly resistant in field trials in Debre Zeit, Ethiopia in 2009 (Olivera et al. 2012), 2014, and 2016 (unpublished data). Thus, PI 192051 is an effective source of resistance not only to race Ug99 but also to other *P. graminis* f. sp. *tritici* races recently observed in Ethiopia which are phylogenetically different from the Ug99 race group. Mapping of *Lr* and *Sr* genes in PI 192051 is ongoing because this genotype seems to carry previously uncharacterized genes in durum cultivars with a broad spectrum of resistance.

**Conclusion**. The objective of the current study was to identify new sources of resistance to leaf rust and stem rust that can be useful to broaden the narrow rust resistance spectrum in durum wheat. Eight durum genotypes from the USDA-NSGC that are mainly landraces and come from different geographical locations were used in the current study. The inheritance study revealed that five of the crosses (Rusty × PI 192051, Divide × PI 244061, Rusty × PI387263, Rusty × PI 209274, and Divide × PI 313096) carried single dominant *Lr* genes effective to *P. triticina* race BBBQJ. In the remaining crosses (Rusty × PI 534304, Rusty × PI 278379, Rusty × PI 195693, and Divide × PI 195693), the inheritance of *Lr* genes was more complex, involving recessive resistance, two genes, or deviation from simple Mendelian inheritance. The leaf rust resistance in seven genotypes used to develop the biparental populations was conferred, at least in part, by genes different from previously mapped genes in durum cultivars The eight genotypes resistant to BBBQJ have resistance to additional *P. triticina* races tested at both the seedling stage in the greenhouse and at the adult stage in field trials. Therefore, more research is needed to verify whether the resistance to different races in each of these genotypes is conferred by the same or different genes. The BSA showed that the *Lr* genes in PI 209274, PI 244061, PI387263, and PI 313096 were mapped to chromosome arms 6BS, 2BS, 6BL, and 6BS, respectively. Further mapping of the likely new or underutilized *Lr* genes using KASP markers narrowed down the genomic regions of the *Lr* genes in PI 244061, PI 387263, and PI 209274. *LrPI387263* mapped to 2.8 cM distal to *KASP_6BL_IWB44753*, *LrPI244061* mapped to 11.5 cM distal to *KASP_2BS_IWB6117*, and *LrPI209274* was flanked by *KASP_6BS_IWB39456* and *KASP_6BS_IWA3298* to a 4.7-cM region. Two of the eight genotypes were also resistant to *P. graminis* f. sp. *tritici* race TTKSK. The resistance in PI 534304 was conferred by a single dominant gene on 6AL, which is most likely *Sr13*. PI 192051 possessed a wide spectrum of resistance to *P. graminis* f. sp. *tritici* races. which could be conferred by an uncharacterized resistance gene in durum germplasm.
